# Overexpression of hippocampal AdipoR1 ameliorates cognitive dysfunction in APP/PS1 mice through inhibiting NLRP3-Mediated neuronal pyroptosis

**DOI:** 10.1016/j.neurot.2026.e01093

**Published:** 2026-10-26

**Authors:** Chunnian Hu, Jing Li, Lin Jiang, Yuning Zhou, Yuhui Deng, Qing Pan, Shun Wang, Wenyu Yang, Lei Zhang, Fenglei Chao, Yanmin Luo, Jing Tang, Qian Xiao, Xin Liang, Yingqiang Qi, Yong Tang, Chunni Zhou

**Affiliations:** a Department of Histology and Embryology, College of Basic Medicine, Chongqing Medical University, Chongqing, China; b Laboratory of Stem Cells and Tissue Engineering, College of Basic Medicine, Chongqing Medical University, Chongqing, China; c Lab Teaching & Management Center, Chongqing Medical University, Chongqing, China; d Department of Physiology, College of Basic Medicine, Chongqing Medical University, Chongqing, China; e Department of Radioactive Medicine, Chongqing Medical University, Chongqing, China; f Department of Pathology, Chongqing Medical University, Chongqing, China

**Keywords:** Alzheimer’s disease, Neuron, Dendritic spine, AdipoR1, NLRP3

## Abstract

Alzheimer’s disease (AD) is a prevalent neurodegenerative disorder primarily characterized by progressive cognitive dysfunction. Substantial loss of hippocampal neurons is closely associated with irreversible cognitive decline; however, the underlying mechanisms have not yet been fully elucidated. In this study, conditionally overexpressing adiponectin receptor 1 (AdipoR1) in the hippocampus of APP/PS1 transgenic AD mice, we found that it significantly improved learning and memory abilities and effectively mitigated the abnormal loss of hippocampal neurons and dendritic spines. AdipoR1 overexpression primarily maintained neuronal numbers through reducing neuronal death rather than promoting neurogenesis, and this protective effect is closely linked to the inhibition of the neuronal NLRP3 inflammasome, leading to a marked reduction in pyroptosis levels within the hippocampus. Our results demonstrated, for the first time, that AdipoR1 exerted a crucial neuroprotective role in AD pathology through modulating the NLRP3-mediated neuroinflammatory pathway, which might offer new insights into the mechanisms of neuronal loss in AD and suggest that the AdipoR1–NLRP3 signaling axis may serve as a potential therapeutic target for early intervention in AD.

## Introduction

Alzheimer’s disease (AD) is a neurodegenerative disorder characterized by progressive cognitive dysfunction as its core clinical feature [1]. Its typical neuropathological hallmarks include extracellular deposition of β-amyloid (Aβ) forming senile plaques, intracellular neurofibrillary tangles resulting from tau protein hyperphosphorylation, and subsequent neuronal loss, synaptic damage, and glial cell activation [2]. The hippocampus, a brain region crucial for learning and memory functions, undergoes pathological changes and functional impairment early in the course of AD [3,4]. Postmortem studies have revealed that severe hippocampal neuronal loss directly correlates with the rapid cognitive decline observed in AD patients [5,6]. However, to date, no effective drugs are available to prevent hippocampal neuronal loss in Alzheimer’s disease.

Hippocampal neuronal loss in both AD rodent models and human AD patients is a multifactorial process involving two interrelated mechanisms: impaired adult hippocampal neurogenesis (AHN) [7,8] and widespread death of mature neurons through various programmed cell death pathways, including apoptosis and pyroptosis [9]. AHN refers to the dynamic process that begins with the proliferation of neural stem/progenitor cells, progresses through differentiation into neurons, and ultimately involves the functional integration of newborn neurons into the existing hippocampal dentate gyrus circuitry via complex maturation and integration processes [10]. Numerous studies have indicated that AHN is significantly reduced in the early stages, and even the prodromal phase in both rodent models of AD and human patients [7,8]. Decreased neuronal proliferation, differentiation, and survival of newborn neurons impair the plasticity and self-repair capacity of hippocampal neural circuits, leading to memory deficits in AD patients [8]. Choi et al. found that synergistically enhancing adult hippocampal neurogenesis and brain-derived neurotrophic factor (BDNF) signaling significantly improved synaptic dysfunction and cognitive deficits in a mouse model of AD, suggesting that restoring processes related to hippocampal neuroplasticity has the potential to reverse cognitive impairment in AD [11]. Furthermore, both optogenetic stimulation [12] to enhance hippocampal neurogenesis and the restoration of miR-132 expression [13] in the hippocampus of AD mice improved AHN deficits and alleviated cognitive impairments, further supporting the critical role of regulating hippocampal neurogenesis in improving AD-related cognitive impairment. On the other hand, various AD-related pathological factors, including Aβ, neuroinflammation, and oxidative stress, activate cell death pathways, driving hippocampal neuron death and ultimately resulting in hippocampal atrophy and neural circuit disruption [9]. The death pathways affecting mature neurons in AD encompass multiple programmed cell death modalities, such as apoptosis, necroptosis, and pyroptosis, among others [14]. Recent studies have emphasized that pyroptosis may act as a crucial driving factor in the pathogenesis of AD patients [15,16]. Pyroptosis is a programmed, highly pro-inflammatory form of cell death characterized by cell swelling, plasma membrane rupture, and the release of immunostimulatory contents and inflammatory mediators such as IL-1β and IL-18 [17,18]. Therefore, strategies aimed at modulating neurogenic processes and/or inhibiting neuronal death hold promise for ameliorating memory impairment in AD.

In recent years, the neuroprotective role of adiponectin receptor 1 (AdipoR1) has garnered increasing attention [19,20]. As a key receptor for adiponectin, AdipoR1 is widely distributed in brain regions most vulnerable to AD pathology, such as the hippocampus and cortex [21], and is highly expressed in neurons [22]. Studies have confirmed that AdipoR1 levels are significantly reduced in the brain tissue of AD patients and animal models [23,24], suggesting its potential involvement in AD pathogenesis. AdipoR1 is not only involved in regulating energy homeostasis [25], but also plays important roles in anti-inflammation, anti-oxidative stress, promoting neuronal survival, and synaptic plasticity [19]. In vitro studies have shown that AdipoRon, a broad agonist of adiponectin receptors, can ameliorate Aβ-induced proliferation deficits in NE-4C neural stem cells via the AdipoR1/AMPK/CREB signaling pathway [26]. Furthermore, neuronal AdipoR1 deficiency exacerbates mitochondrial damage and oxidative stress-induced neuronal death following traumatic brain injury in mice [20]. Additionally, adiponectin and its homologs can influence the activation of pyroptosis-related factors such as NLRP3 and IL-1β in chondrocytes and epithelial cells via AdipoR1, reducing inflammatory responses [27]. The NLRP3 inflammasome is a core component of the canonical pyroptosis signaling pathway [28]. Previous studies confirmed that the NLRP3 inflammasome and its mediated pyroptosis were activated in the hippocampus of AD patients and animal models, and they were closely linked to cognitive impairment [29,30]. These studies suggested that AdipoR1 may play a significant role in neurogenesis and/or pyroptosis. However, whether AdipoR1 in the central nervous system, particularly in the context of AD, can improve spatial learning and memory deficits in AD mice through inhibiting NLRP3-mediated neuronal pyroptosis and/or enhancing adult hippocampal neurogenesis remains unclear.

Based on this background, the present study aimed to systematically investigate whether AdipoR1 improves cognitive function in APP/PS1 transgenic mice through regulating hippocampal neuronal loss. AdipoR1 was conditionally overexpressed in the hippocampus, and spatial learning and memory function was assessed using the Morris water maze. Subsequently, stereological analyses and immunofluorescence techniques were employed to investigate the mechanisms underlying AdipoR1-mediated hippocampal neuronal loss.

## Materials and Methods

### Animals

APP/PS1 double transgenic [B6C3-Tg (APPswe, PSEN1dE9) 85Dbo/NJU] breeding mice used in this study were purchased from the Model Animal Research Institute of Nanjing University (License No.: SCXK-2010-0001) and housed in the Specific Pathogen Free (SPF) Experimental Animal Center of Chongqing Medical University. Animals were maintained under standard conditions: temperature (22 ± 2 °C), humidity (50 ± 10%), and a 12-h light/dark cycle, with *ad libitum* access to food and water. Heterozygous APP/PS1 mice were bred with C57BL/6 J mice to obtain offspring. Littermates of APP/PS1 transgenic mice were used. Genotypes were accurately identified by PCR amplification and agarose gel electrophoresis using tail tip samples. Mice positive for both APP (350 bp) and PS1 (608 bp) gene-specific bands were identified as APP/PS1 double transgenic mice; littermate control samples showing no target bands were confirmed as wild-type (WT) mice. All animal protocols were approved by the Institutional Animal Care and Use Committee at Chongqing Medical University (approval No. 2021077).

### Hippocampal stereotaxic injection and BrdU administration

Previous studies have demonstrated that 8-month-old APP/PS1 mice are in an early stage of pathological progression, characterized by cognitive deficits, synaptic damage accompanied by mild neuronal loss, and progressively increasing cerebral amyloid plaque burden [31,32]. Meanwhile, it has been reported that the levels of soluble and insoluble Aβ40 and Aβ42 in the brains of mice at this age remain modifiable and reversible [33]. Therefore, this stage has not yet progressed to a phase of extensive, irreversible pathological changes, resembling the preclinical or early stage of AD. Combined with our previous findings that 10-month-old APP/PS1 mice already exhibit detectable Aβ plaque deposition, extensive neuronal loss across hippocampal subregions, and impaired spatial learning and memory [33,34], this study selected 8-month-old APP/PS1 transgenic mice to determine whether AdipoR1 restoration could ameliorate AD-related deficits and attenuate disease progression at an early pathological stage. Forty-five 8-month-old male mice (15 wild-type and 30 APP/PS1 transgenic) were divided into three groups (n = 15 per group): wild-type + control virus (WT + Ctrl), APP/PS1 + control virus (AD + Ctrl), and APP/PS1 + AdipoR1 overexpression virus (AD + OvAdipoR1). Control adeno-associated virus (AAV-NC) or AAV targeting AdipoR1 overexpression (AAV-AdipoR1) was bilaterally injected into the hippocampus via stereotaxic injection. The recombinant adeno-associated virus (AAV) vector was engineered to encode green fluorescent protein (GFP) as a reporter gene, enabling direct visualization of viral injection sites and assessment of transduction efficiency and expression distribution across hippocampal subregions via confocal microscopy [35]. The transcript ID of AdipoR1 used to generate the overexpression adeno-associated vector is NM_001306069.1. The empty vector was GL3001 pcAAV-CMV-EGFP-P2A-MCS-3FLAG-WPRE. The vector we constructed was named pcAAV-CMV-EGFP-P2A-AdipoR1-3xFLAG-WPRET. AAVs were purchased from OBiO Technology Co., Ltd. (Shanghai, China). The stereotactic injection method was consistent with that previously described by our group [36]. According to the characteristics of AAV vectors and relevant literature, the target gene typically begins to express 1–2 weeks post-injection and reaches a stable level at approximately 3–4 weeks [37]. Two weeks after stereotaxic injection, all mice received intraperitoneal injections of 5-bromo-2′-deoxyuridine (BrdU; Sigma-Aldrich, St. Louis, MO, USA) (50 mg/kg/day) for 5 consecutive days. Behavioral tests were performed two months after virus injection.

### Morris water maze test

Two months after injection, spatial learning and memory abilities were assessed using the Morris water maze task conducted over 7 consecutive days. In visible platform training (Day 1), the escape platform was elevated 0.5–1 cm above the water surface to ensure visibility. Following a 15-s habituation period on the platform, each mouse was released into the pool from the two quadrants farthest from the platform. Swimming speed and escape latency were recorded for each trial (cut-off time: 60 s). Animals that failed to locate the platform within 60 s were gently guided to it and allowed to remain for 15 s. In hidden platform training (Days 2–6), the platform was submerged below the water surface. Mice received four training trials per day initiated from different quadrants in a randomized order. Escape latency was recorded for each trial. In probe trial (Day 7), the platform was removed from the pool. Mice were released from the two quadrants farthest from the target quadrant (former platform location), and a single 60-s probe trial was conducted. Spatial memory retention was evaluated by recording the time spent in the target quadrant, the ratio of distance traveled in the target quadrant to total distance, and the number of platform crossings.

### Quantitative real-time PCR (RT-qPCR)

Total RNA was extracted from hippocampal tissue using MagZol Reagent according to the manufacturer’s protocol. RNA was reverse-transcribed into cDNA using the Evo M-MLV RT Mix Kit with gDNA Clean for qPCR Ver.2 (Accurate Biology, Hunan, China). Real-time quantitative PCR was performed on a Bio-Rad CFX96 system using 2 × SYBR Green qPCR Master Mix (Selleck, Houston, TX, USA).

### Western blotting

Hippocampal tissue was lysed in RIPA buffer (Beyotime Biotechnology, Shanghai, China) containing protease and phosphatase inhibitors. Lysates were centrifuged at 12,000×*g* for 10 min at 4 °C to remove insoluble material. Protein concentration was determined using the BCA Protein Assay Kit (Beyotime Biotechnology). Proteins were denatured in 5 × SDS-PAGE loading buffer at 95 °C for 10 min, separated on 10% PAGE gels (Yeasen Biotechnology, Shanghai, China), and transferred to polyvinylidene difluoride (PVDF) membranes. Membranes were blocked in Quick Block solution (Beyotime Biotechnology) for 1 h at room temperature, then incubated overnight at 4 °C with primary antibodies. After washing with Tris-buffered saline with Tween 20 (TBST), membranes were incubated with horseradish peroxidase (HRP)-conjugated secondary antibodies for 1 h at room temperature. Bands were visualized using enhanced chemiluminescence substrate, and densitometry was performed using ImageJ. Primary antibodies used: rabbit anti-AdipoR1 (1:1000; Novus Biologicals, Centennial, CO, USA; NBP2-67631), rabbit anti-AdipoR2 (1:1000; Santa Cruz Biotechnology, Dallas, TX, USA; sc-514,045), rabbit anti-NeuN (1:1000; Abcam, Cambridge, UK; EPR12763), chicken anti-NeuN (1:500; EMD Millipore, Burlington, MA, USA; ABN91), rat anti-BrdU (1:500; Abcam; AB6326), rabbit anti-NLRP3 (1:1000; Novus Biologicals), mouse anti-Caspase 1 (1:1000; Santa Cruz Biotechnology; sc-392,736), mouse anti-GSDMD (C-terminus) (1:1000; Santa Cruz Biotechnology; sc-393,656), mouse anti-IL-1β (1:1000; Santa Cruz Biotechnology; sc-52012), mouse anti-IL-18 (1:1000; Wanleibio, Shenyang, China; WL01127), rabbit anti-ASC (1:1000; Cell Signaling Technology, Danvers, MA, USA; 67,824), mouse anti-β-Tubulin (1:1000; Abmart, Shanghai, China; M20005F), mouse anti-β-actin (1:1000; Abmart; T40104). Secondary antibodies: HRP-conjugated rabbit IgG (1:1000; Abbkine Scientific, Wuhan, China; A21020), HRP-conjugated mouse IgG (1:1000; Abbkine Scientific; A21010).

### Processing of brain tissue

Randomly selected mice were deeply anesthetized with 1% sodium pentobarbital and transcardially perfused with heparinized saline, followed by 4% paraformaldehyde. Brains were removed, post-fixed, and cryoprotected in graded sucrose solutions (10%, 20%, 30%) in 0.1 M PBS. The preserved brain tissue was carefully divided into left and right hemispheres. One randomly selected hemisphere was then cut into 30-μm-thick serial sections and the other hemisphere into 50-μm-thick serial sections using a Leica cryosectioner (Leica CM1950, Leica, Germany). The 30-μm-thick sections were sampled in fractions of 1/20 for immunofluorescence staining, and the 50-μm-thick sections were sampled in fractions of 1/5 for immunohistochemical staining. The remaining slices were rinsed with PBS rinse to remove the OCT embedding compound, sections were stored in 75% ethanol at −20 °C.

Additionally, mice were randomly selected and anesthetized using the method described above. Fresh bilateral hippocampal tissues were dissected, collected, and stored at −80 °C for subsequent RNA and protein extraction, followed by qPCR and Western blotting analyses.

### Spinophilin immunogold staining

One set of 50-μm hippocampal sections from 5 randomly selected mice per group was used for spinophilin immunogold staining. Sections were rinsed in 0.01 M PBS and Triton-Tween buffer, blocked for 2 h at 37 °C, and incubated with anti-spinophilin antibody (in Triton-Tween buffer) for 72 h at 4 °C. After washing, sections were incubated with gold-conjugated secondary antibody for 3 h at 37 °C, fixed in 2% glutaraldehyde for 8 min, and washed. Silver enhancement was performed for 30 min in the dark. Sections were mounted, developed with DAB, counterstained with hematoxylin, dehydrated through an ethanol gradient, cleared in xylene, and mounted with neutral balsam.

### Nissl staining

One set of 50-μm hippocampal sections from 5 randomly selected mice per group was used for Nissl staining. Sections were mounted, rinsed in PBS, permeabilized with 3% Triton X-100 in PBS for 2 h, and stained with preheated 0.04% toluidine blue solution at 57 °C for 50 min. After rinsing, sections were differentiated in 95% ethanol, dehydrated in absolute ethanol, cleared in xylene, and coverslipped.

### Stereological analyses

The total numbers of Nissl-stained neurons and spinophilin-positive (Sp^+^) dendritic spines in the hippocampal subregions (CA1, CA3, and dentate gyrus [DG]) were estimated in an unbiased manner using the optical fractionator method combined with systematic uniform random sampling (SURS) [38,39]. Coronal sections were collected along the rostro-caudal axis at a fixed interval using SURS, yielding a section sampling fraction (*s*sf) of 1/5. Hippocampal subregions were delineated under a 10 × objective, and all counting was performed under a 100 × oil-immersion objective (Fig. 1a and b). Neuron counting: Neuronal somata stained with toluidine blue (Nissl staining) were counted using an unbiased counting frame of 90 μm × 65 μm. The area sampling fraction (*a*sf) was set to 1% (0.01). To avoid sectioning artifacts and lost caps, a 3-μm guard zone was set at the top surface of each section. Neurons were counted through an optical height (*h*) of 15 μm below the guard zone. The height sampling fraction (hs*f*) was calculated as the disector height (*h*) divided by the mean section thickness (*t*) for each individual mouse: hsf = *h*/*t*. The total number of neurons (*N*<sub>) in each subregion was calculated according to the optical fractionator formula:N<sub> = ΣQ⁻<sub> · (1/*s*sf) · (1/*a*sf) · (1/hs*f*). Dendritic spine counting: Sp^+^ dendritic spines were identified by spinophilin immunofluorescence (or immunoperoxidase) staining and counted using an unbiased counting frame with an area of 4.75 μm^2^. The area sampling fraction (*a*sf) was set to 0.02% (2 × 10^−4^). A 3-μm guard zone was applied at the top surface, and Sp^+^ spines were counted through an optical height (*h*) of 10 μm below the guard zone. The height sampling fraction (hs*f*) was calculated as *h*/*t*. The total number of Sp ^+^ spines (*N*<sub>) in each subregion was calculated as [36,38]: N<sub> = ΣQ⁻<sub> · (1/*s*sf) · (1/*a*sf) · (1/hs*f*). Volume was estimated using toluidine blue Nissl-stained brain sections without rinsing, to avoid volume artifacts caused by tissue shrinkage during the washing procedure. Volume was estimated using the Cavalieri principle: V = ΣP × a(p) × t × 5 × 2, where ΣP is the total number of points hitting the region, a(p) is the area per point, and t is the section thickness (Fig. 1c and d).

**Fig. 1:**
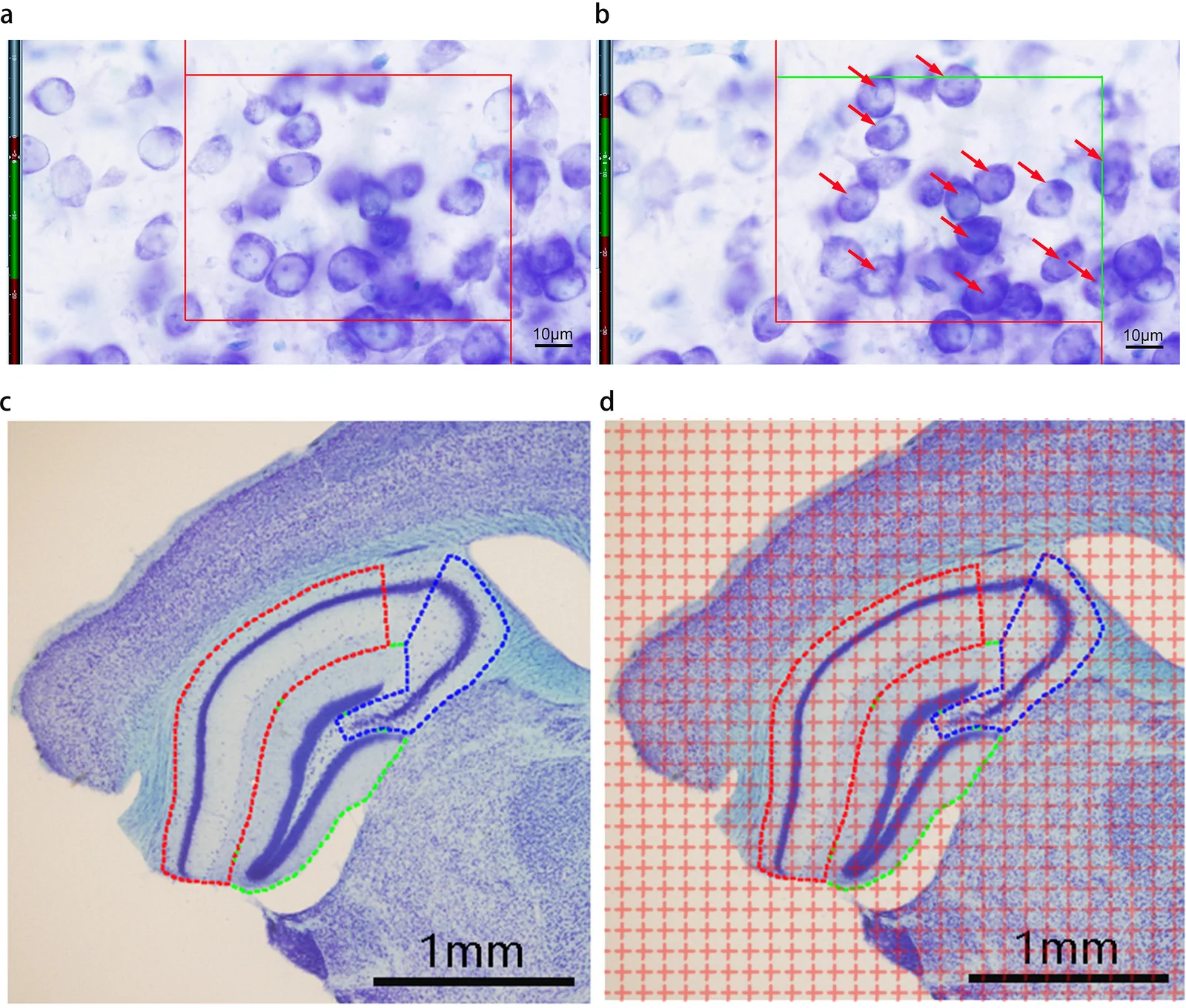
Schematic diagram of hippocampal subregions and stereological counting frames. (a–b) Two representative images show the unbiased counting frame. The red line of the frame and its extension represent the exclusion lines, and the green line represents the inclusion line. Neuronal nuclei are counted when they first come into focus if they are completely inside the counting frame or partly inside and touching the inclusion (green) line. Red arrows indicate all neurons counted within this field of view. Scale bar = 20 μm. (c) Illustration of hippocampal subregion volume estimation. Green: DG, Red: CA1, Blue: CA3, scale bar = 1 mm. (d) Equidistant test points are superimposed on the contours of hippocampal subregions, and the points intersecting each subregion are counted to estimate the volume according to the Cavalieri principle. Scale bar = 1 mm.

### Immunofluorescence staining

Free-floating 30-μm sections were washed in PBS, permeabilized in Triton-Tween buffer, and subjected to antigen retrieval in citrate buffer (95 °C, 20 min). After blocking (2 h, 37 °C), sections were incubated overnight at 4 °C with primary antibodies: AdipoR1/NeuN; NLRP3/NeuN; GSDMD/NeuN; BrdU/doublecortin (DCX)/NeuN. After washing, sections were incubated with appropriate fluorescent secondary antibodies for 2 h at 37 °C in the dark. Nuclei were counterstained with 4′,6-diamidino-2-phenylindole (DAPI). Sections were mounted with anti-fade mounting medium.

### Statistical analysis

Statistical analyses were performed using SPSS v.27.0. Data are presented as mean ± standard deviation (SD). Outliers were defined as values exceeding mean ± 2 SD and were excluded. Water maze escape latency data (days 2–6) were analyzed using repeated-measures ANOVA. For comparisons among three groups, one-way ANOVA followed by LSD post-hoc test was used for normally distributed data. Non-normally distributed data were analyzed using the Kruskal-Wallis H test. Statistical significance was set at *P* < 0.05.

## Results

### Hippocampal AdipoR1 expression was decreased in APP/PS1 mice, and overexpression of hippocampal AdipoR1 improved learning and memory

Immunofluorescence revealed significantly reduced AdipoR1 expression in hippocampal neurons of AD mice (Fig. 2a). RT-qPCR and Western blotting further confirmed that both mRNA and protein levels of AdipoR1 were significantly decreased in the hippocampus of AD+Ctrl mice compared to WT+C controls (*P* = 0.005, *P* < 0.001; Fig. 2b-d).

**Fig. 2:**
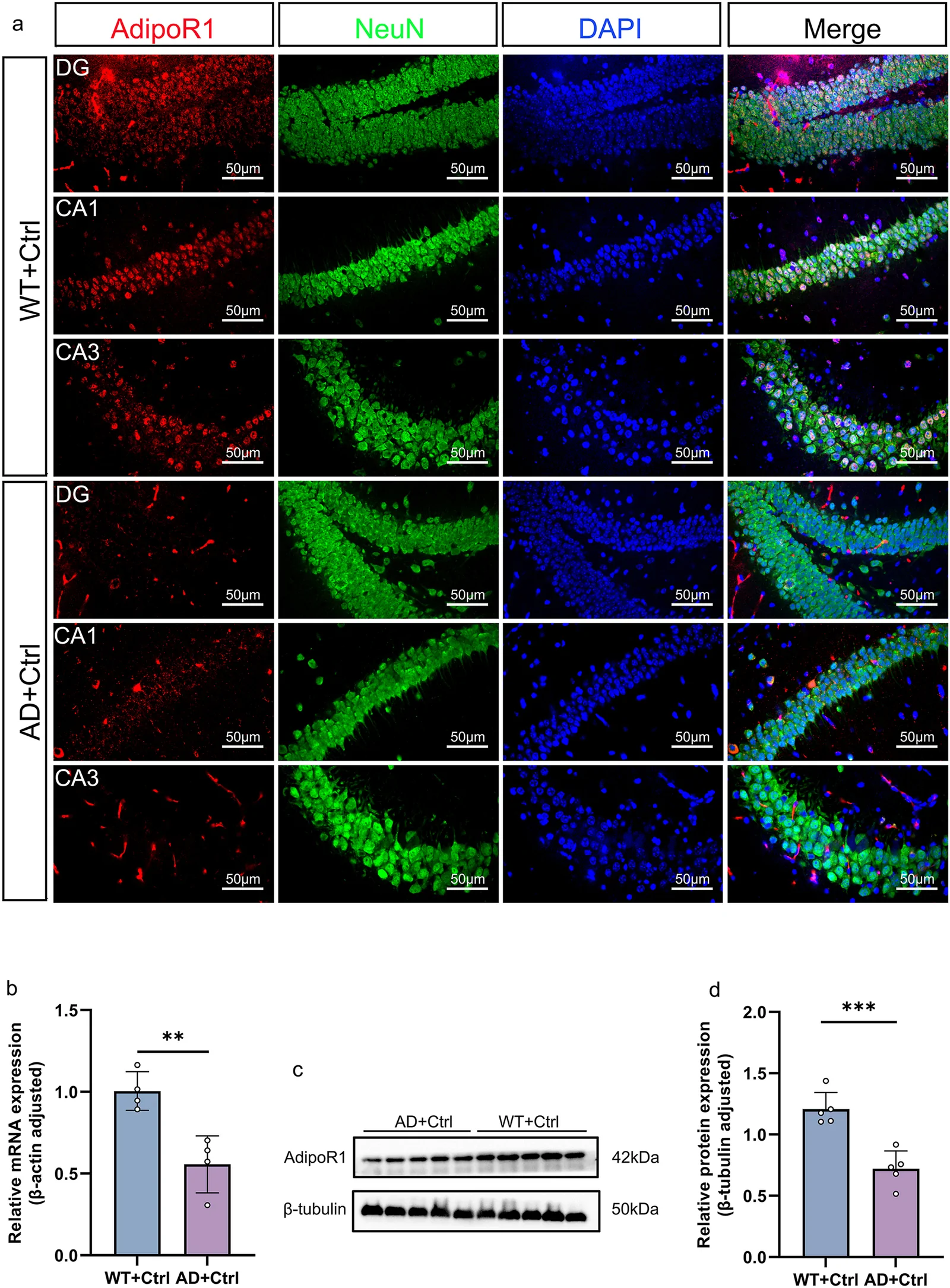
AdipoR1 expression is decreased in the hippocampus of APP/PS1 mice. (a) Immunofluorescence staining for NeuN (green), AdipoR1 (red) and DAPI (blue) in the DG, CA1, and CA3 subregions of the hippocampus in WT + Ctrl and AD + Ctrl mice. (b) Relative mRNA levels of AdipoR1 in the hippocampus of WT + Ctrl and AD + Ctrl mice (n = 4 per group). (c–d) Quantification of AdipoR1 protein expression levels in the hippocampus (n = 5 per group). ∗*P* < 0.05; ∗∗*P* < 0.01; ∗∗∗*P* < 0.001.

To investigate the role of AdipoR1 in AD pathogenesis, we used adeno-associated virus combined with stereotaxic injection to specifically overexpress AdipoR1 bilaterally in the hippocampus of AD mice. Fluorescence imaging revealed that viral expression was predominantly confined to the hippocampal formation and extended along the dorsoventral axis, substantially covering the major hippocampal subregions, including CA1, CA3, and DG (Fig. 3a). Immunofluorescence co-staining of AdipoR1 and NeuN confirmed AdipoR1 expression in the hippocampal cell layers of AD mice (Fig. 3b). RT-qPCR showed significantly increased AdipoR1 mRNA expression in the hippocampus of AD + OvAdipoR1 mice compared to AD + Ctrl mice (*P* < 0.001; Fig. 3c). Western blotting demonstrated that AdipoR1 protein level was significantly lower in AD + Ctrl mice compared to WT + Ctrl mice, and significantly higher in AD + OvAdipoR1 mice compared to AD + Ctrl mice (*P* = 0.003, *P* = 0.039; Fig. 3d and e), confirming effective overexpression. Morris water maze analyses were then performed. On day 1 (visible platform), no significant differences in escape latency were observed among the three groups, indicating no impairment in vision or swimming ability (*P* = 0.350; Fig. 4c). Compared to WT + Ctrl mice, AD + Ctrl mice showed significantly longer escape latencies on days 2, 3, 4, 5, and 6 (*P* < 0.001; Fig. 4d). Compared to AD + Ctrl mice, AD + OvAdipoR1 mice exhibited significantly shorter escape latencies on these training days (*P* < 0.001; Fig. 4d). In the probe test on day 7, AD + Ctrl mice displayed significantly fewer platform crossings, reduced time spent in the target quadrant, and a decreased distance ratio in the target quadrant compared to WT + Ctrl mice (*P* = 0.009, *P* = 0.019, *P* = 0.029; Fig. 4e-g). Conversely, AD + OvAdipoR1 mice showed significant improvements in all these parameters compared to AD + Ctrl mice (*P* = 0.031, *P* = 0.013, *P* = 0.015; Fig. 4e-g). Open field test results revealed that, in the absence of significant differences in locomotor activity among the three groups (*P* = 0.287, *P* = 0.415; Supplementary Fig. 1b), the AD + Ctrl mice exhibited marked reductions in both the percentage of distance traveled and the percentage of time spent in the central zone (*P* = 0.031, *P* = 0.049; Supplementary Fig. 1c–d), indicating the presence of anxiety-like behavior. Notably, AdipoR1 overexpression showed a trend toward alleviating anxiety-like behavior compared with AD + Ctrl mice, although the differences did not reach statistical significance (*P* = 0.077, *P* = 0.146; Supplementary Fig. 1c–d). These results indicated that hippocampal AdipoR1 was crucial for cognitive function in AD.

**Fig. 3:**
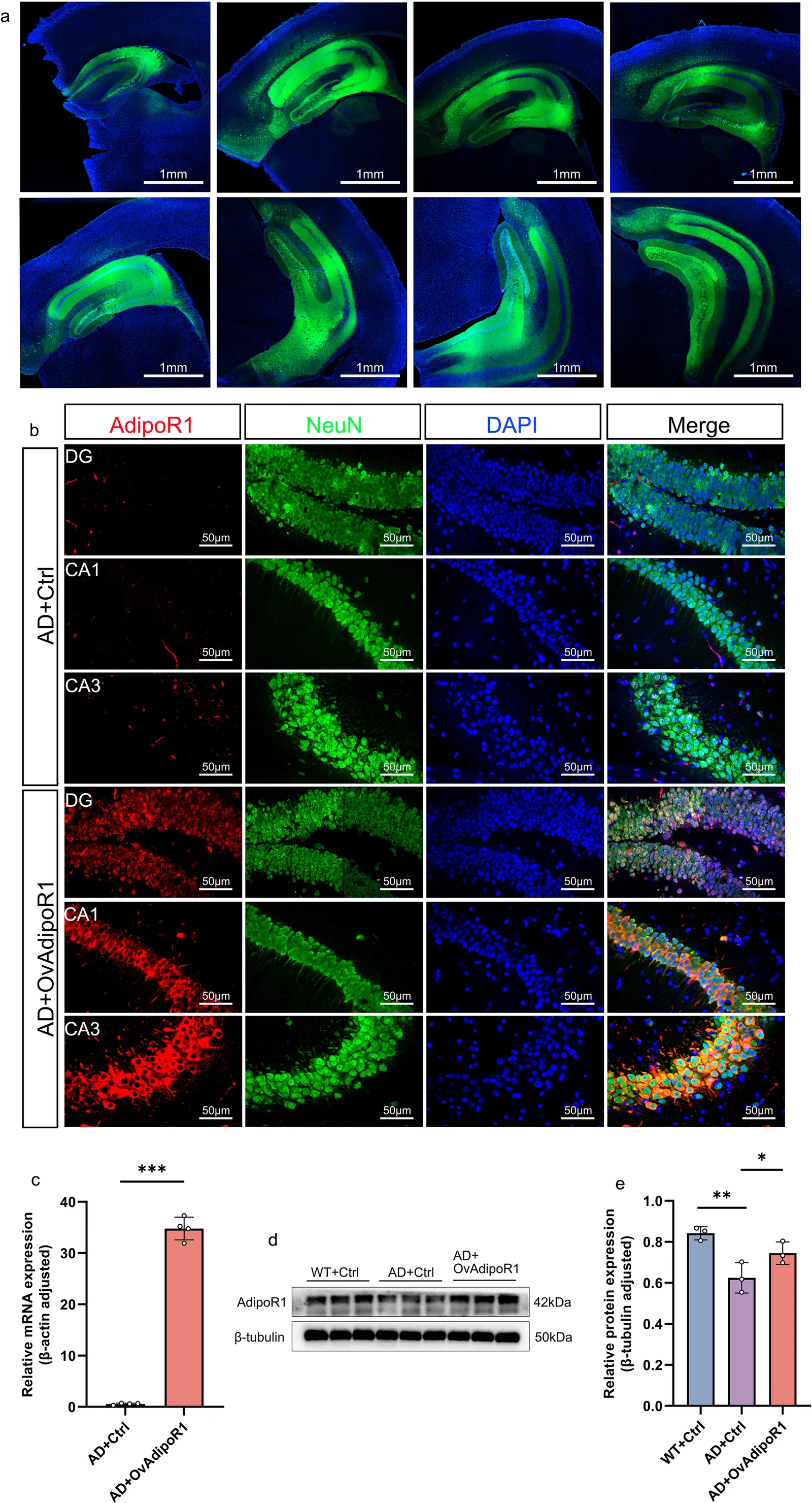
Establishment of a hippocampus-specific AdipoR1 overexpression model in APP/PS1 mice. (a) Representative serial hippocampal coronal sections at different rostrocaudal levels showing EGFP expression in the mouse brain, indicating successful AAV transduction of the hippocampus. Scale bar = 1 mm. (b) Immunofluorescence staining schematic diagrams for NeuN (green), AdipoR1 (red) and DAPI (blue) in the DG, CA1, and CA3 subregions of the hippocampus in AD + Ctrl and AD + OvAdipoR1 mice. (c) Relative mRNA levels of AdipoR1 in the hippocampus of AD + Ctrl and AD + OvAdipoR1 mice (n = 4 per group). (d–e) Quantification of AdipoR1 protein expression levels of WT + Ctrl, AD + Ctrl and AD + OvAdipoR1 mice in the hippocampus (n = 3 per group). ∗*P* < 0.05; ∗∗*P* < 0.01; ∗∗∗*P* < 0.001.

**Fig. 4:**
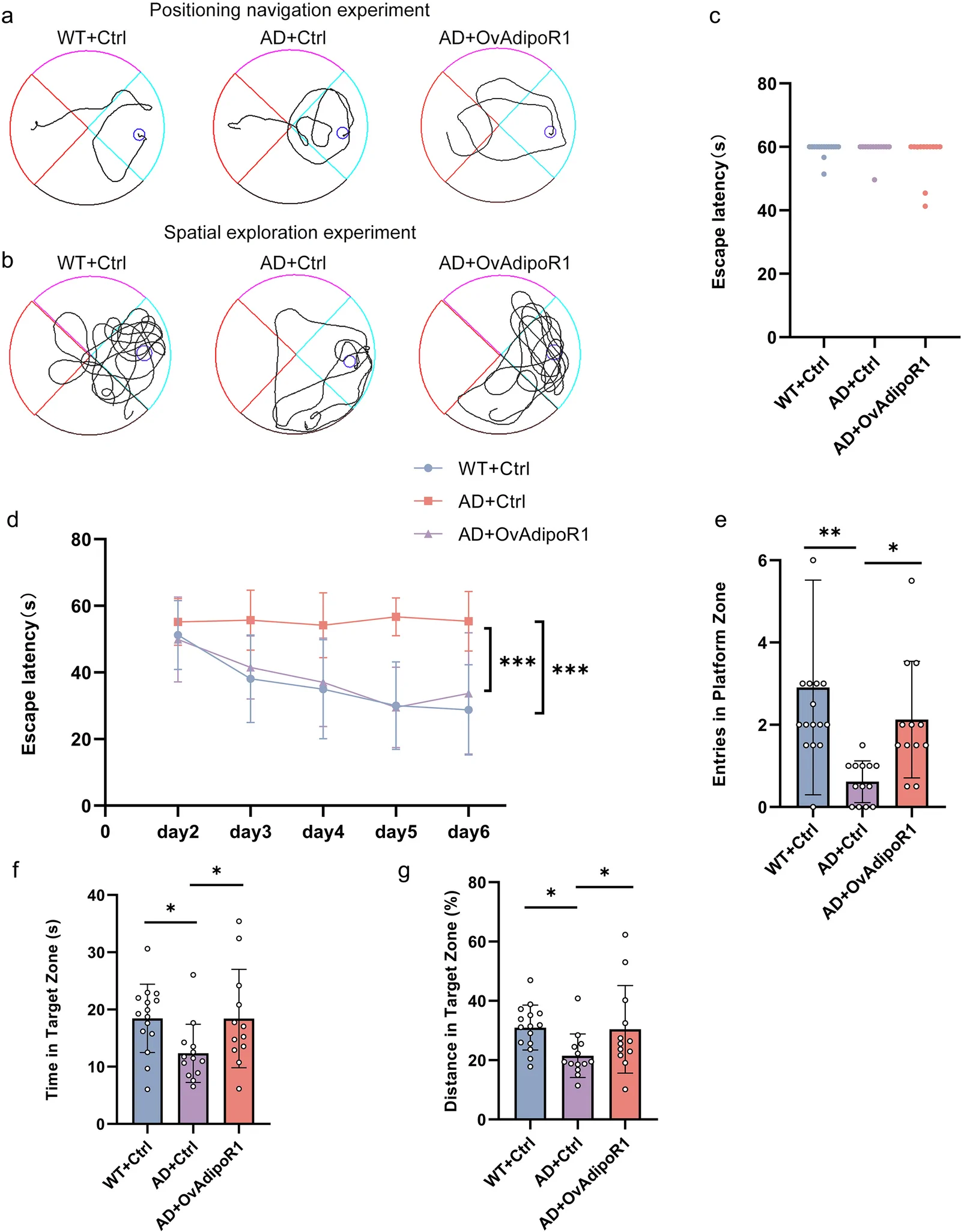
AdipoR1 overexpression improves spatial learning and memory in APP/PS1 mice. (a) Representative swimming trajectories during hidden platform training. (b) Representative swimming trajectories during the probe test (day 7). (c) Visible platform test (mean ± SD). (d) Escape latency on days 2–6; each point represents the group mean (mean ± SD). (e) Number of platform crossings during the probe test (mean ± SD). (f) Time spent in the target quadrant during the probe test (mean ± SD). (g) Percentage of distance swum in the target quadrant during the probe test (mean ± SD).

### Overexpression of hippocampal AdipoR1 ameliorated hippocampal volume atrophy and reduced hippocampal Aβ deposition in APP/PS1 mice

Next, we investigated the effect of AdipoR1 on the volume of hippocampal subregions and total hippocampus. Compared to WT + Ctrl mice, AD + Ctrl mice exhibited significantly reduced volumes of the CA3 region, DG region, and total hippocampus (*P* < 0.001, *P* = 0.036, *P* = 0.007; Fig. 5b), while CA1 volume showed no significant difference (*P* = 0.134; Fig. 5b). Overexpression of hippocampal AdipoR1 significantly increased the volumes of CA1, CA3, DG, and total hippocampus in AD + OvAdipoR1 mice compared to AD + Ctrl mice (*P* = 0.017, *P* = 0.002, *P* = 0.014, *P* = 0.008; Fig. 5b). Hippocampal Aβ load is shown in Fig. 5d. We found that the density of Aβ plaques was significantly reduced in the CA1, CA3, and DG regions of the hippocampus in AD + OvAdipoR1 mice compared to AD + Ctrl mice (*P* = 0.001, *P* = 0.043, *P* = 0.038; Fig. 5d).

**Fig. 5:**
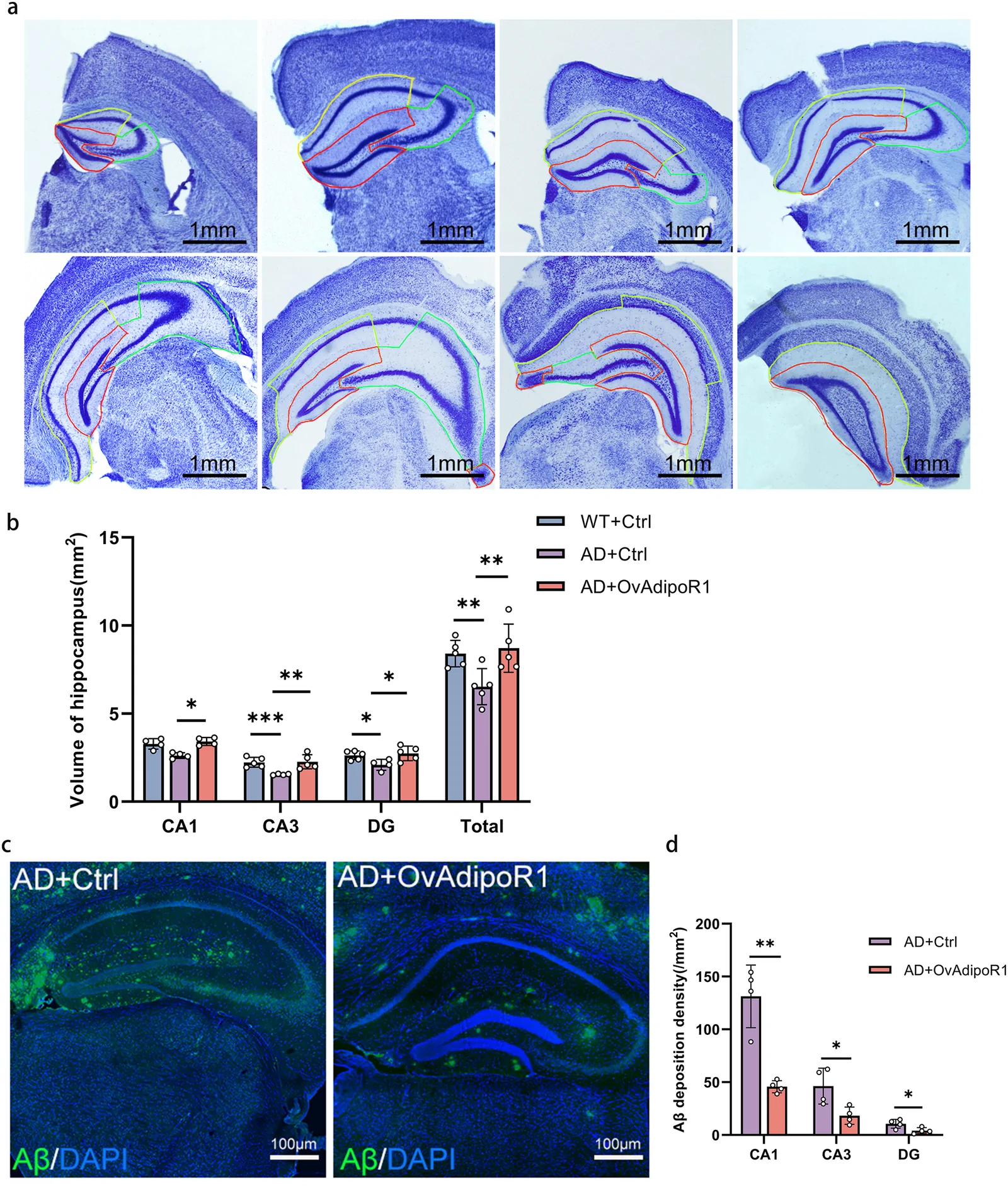
AdipoR1 overexpression ameliorates hippocampal atrophy and Aβ plaque deposition in APP/PS1 mice. (a) Schematic diagram showing hippocampal subregions (DG, CA1, and CA3) across serial coronal sections at different rostrocaudal levels, scale bar = 1 mm. (b) Stereological results of hippocampal volumes in the three groups (mean ± SD). (c) Representative images of immunofluorescence staining for DAPI (blue) and Aβ (green) in hippocampal subregions, scale bar = 100 μm. (d) Aβ plaque density in the hippocampal DG, CA1, and CA3 subregions is quantified by immunofluorescence staining in AD + Ctrl and AD + OvAdipoR1 mice (mean ± SD). ∗*P* < 0.05; ∗∗*P* < 0.01; ∗∗∗*P* < 0.001.

### Overexpression of AdipoR1 ameliorated the loss of hippocampal neurons and dendritic spines in APP/PS1 mice

We used an immunohistochemical technique and a stereological method to precisely quantify the number of neurons and dendritic spines in hippocampal subregions in order to determine whether the cognitive recovery induced by hippocampal AdipoR1 overexpression was associated with changes in these parameters. Representative images of Nissl-stained neurons and immunogold-stained spinophilin-positive dendritic spines in hippocampal subregions are shown in Fig. 6a and c, respectively. The sampling met the criteria, as the observed variance of the individual estimate (OCE^2^) was much <50% of the observed interindividual variance (OCV^2^) (Table 1, Table 2). Compared to WT + Ctrl mice, AD + Ctrl mice showed a significant decrease in the number of neurons in the CA1, CA3, and DG regions (*P* = 0.041, *P* = 0.001, *P* = 0.008; Fig. 6b) and a significant decrease in the number of dendritic spines (*P* < 0.001, *P* = 0.003, *P* = 0.004; Fig. 6d) in the CA1, CA3, and DG regions. Compared to AD + Ctrl mice, AD + OvAdipoR1 mice exhibited a significant recovery in neuronal number in the DG, CA1, and CA3 regions (*P* = 0.015, *P* = 0.038, *P* = 0.032; Fig. 6b) and a significant amelioration of dendritic spine loss (*P* < 0.001, *P* = 0.019, *P* = 0.026; Fig. 6d). These findings suggested that AdipoR1 overexpression can significantly reverse the loss of hippocampal neurons and dendritic spines in AD mice.

**Table 1:** Optical fractionator analysis of hippocampal neuronal number in WT + Ctrl, AD + Ctrl, and AD + OvAdipoR1 mice. Note: Mean, standard deviation (SD), observed coefficient of variation (OCV), and observed coefficient of error (OCE) for stereological sampling. All values in the list are rounded to three decimal places.

		WT + Ctrl	AD + Ctrl	AD + OvAdipoR1
		Neurons	Neurons	Neurons
DG	Mean	1032.750	849.200	983.700
	SD	97.933	76.150	113.295
	OCV	0.095	0.090	0.115
	OCE	0.031	0.034	0.032
	OCE^2^/OCV^2^	0.108	0.147	0.077
CA1	Mean	395.188	270.800	433.050
	SD	92.233	97.825	67.761
	OCV	0.233	0.361	0.156
	OCE	0.051	0.063	0.049
	OCE^2^/OCV^2^	0.049	0.031	0.096
CA3	Mean	379.125	267.800	320.850
	SD	34.411	83.091	42.681
	OCV	0.091	0.310	0.133
	OCE	0.052	0.063	0.056
	OCE^2^/OCV^2^	0.322	0.042	0.179

**Table 2:** Optical fractionator analysis of hippocampal dendritic spine number in WT + Ctrl, AD + Ctrl, and AD + OvAdipoR1 mice. Note: Mean, standard deviation (SD), observed coefficient of variation (OCV), and observed coefficient of error (OCE) for stereological sampling. All values in the list are rounded to three decimal places.

		WT + Ctrl	AD + Ctrl	AD + OvAdipoR1
		SP^+^	SP^+^	SP^+^
DG	Mean	1281.833	967.000	1172.000
	SD	122.975	70.484	207.212
	OCV	0.096	0.073	0.177
	OCE	0.028	0.032	0.030
	OCE^2^/OCV^2^	0.085	0.196	0.028
CA1	Mean	1711.667	1226.167	1678.400
	SD	215.714	165.264	238.309
	OCV	0.126	0.135	0.142
	OCE	0.024	0.029	0.025
	OCE^2^/OCV^2^	0.037	0.046	0.030
CA3	Mean	1306.500	1049.333	1242.000
	SD	177.066	106.899	156.506
	OCV	0.136	0.102	0.126
	OCE	0.028	0.031	0.029
	OCE^2^/OCV^2^	0.042	0.093	0.051

**Fig. 6:**
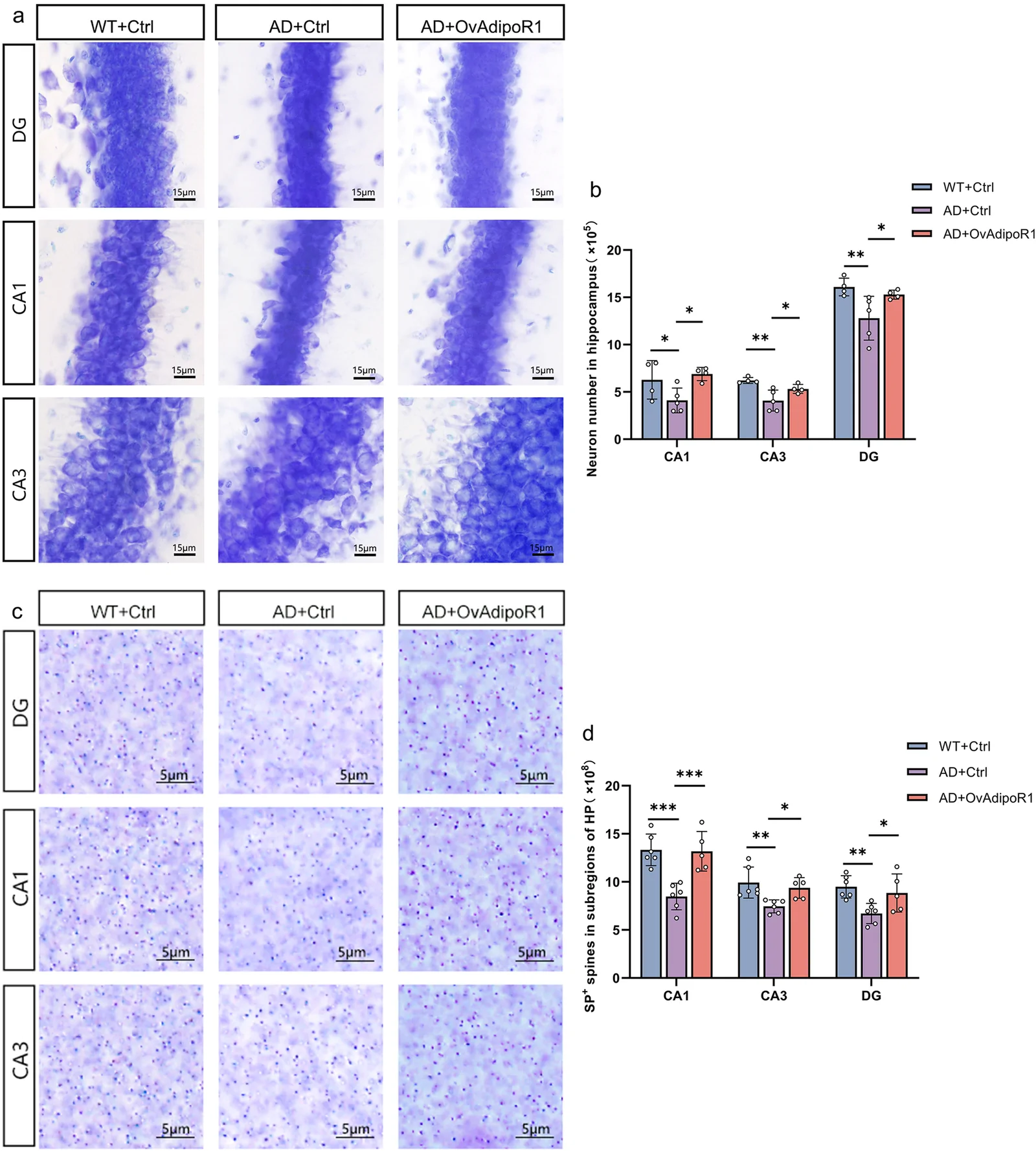
Effects of AdipoR1 overexpression on hippocampal neuron and dendritic spine numbers in APP/PS1 mice. (a) Representative images of Nissl staining (toluidine blue) showing the neuronal cell layers in the hippocampal DG, CA1, and CA3 subregions of WT + Ctrl, AD + Ctrl, and AD + OvAdipoR1 mice, captured under a 100 × oil-immersion objective of a stereology microscope. Scale bar = 15 μm. (b) The number of neurons in the hippocampal DG, CA1, and CA3 subregions is estimated by stereology in WT + Ctrl, AD + Ctrl, and AD + OvAdipoR1 mice (n = 5 per group). (c) Representative spinophilin-immunogold stained images showing dendritic spines in the hippocampal DG, CA1, and CA3 subregions of WT + Ctrl, AD + Ctrl, and AD + OvAdipoR1 mice, captured under a 100 × oil-immersion objective on a stereological microscope. Scale bar = 5 μm. (d) The number of dendritic spines in the hippocampal DG, CA1, and CA3 subregions is estimated by stereology in WT + Ctrl, AD + Ctrl, and AD + OvAdipoR1 mice (n = 6 per group). ∗*P* < 0.05; ∗∗*P* < 0.01; ∗∗∗*P* < 0.001.

### Overexpression of hippocampal AdipoR1 did not affect adult hippocampal neurogenesis in APP/PS1 mice

To further elucidate the effect of AdipoR1 overexpression on different stages of adult hippocampal neurogenesis, we used immunofluorescence to identify and quantify newborn neurons at distinct maturation stages. BrdU^+^/DCX^+^/NeuN^−^ cells were defined as immature newborn neurons, BrdU^+^/DCX^−^/NeuN^+^ cells as mature newborn neurons, and BrdU^+^/DCX^+^/NeuN^+^ cells as a transitional population (Fig. 7a). Compared to WT + Ctrl mice, AD + Ctrl mice exhibited significantly reduced densities of BrdU^+^/DCX^+^/NeuN^+^, BrdU^+^/DCX^−^/NeuN^+^, and BrdU^+^/DCX^+^/NeuN^−^ cells in the DG region (*P* = 0.038, *P* = 0.002, *P* = 0.015; Fig. 7c-e). However, no significant differences were observed in the densities of these three cell populations between AD + OvAdipoR1 and AD + Ctrl mice (*P* = 0.232, *P* = 0.144, *P* = 0.725; Fig. 7c-e).

**Fig. 7:**
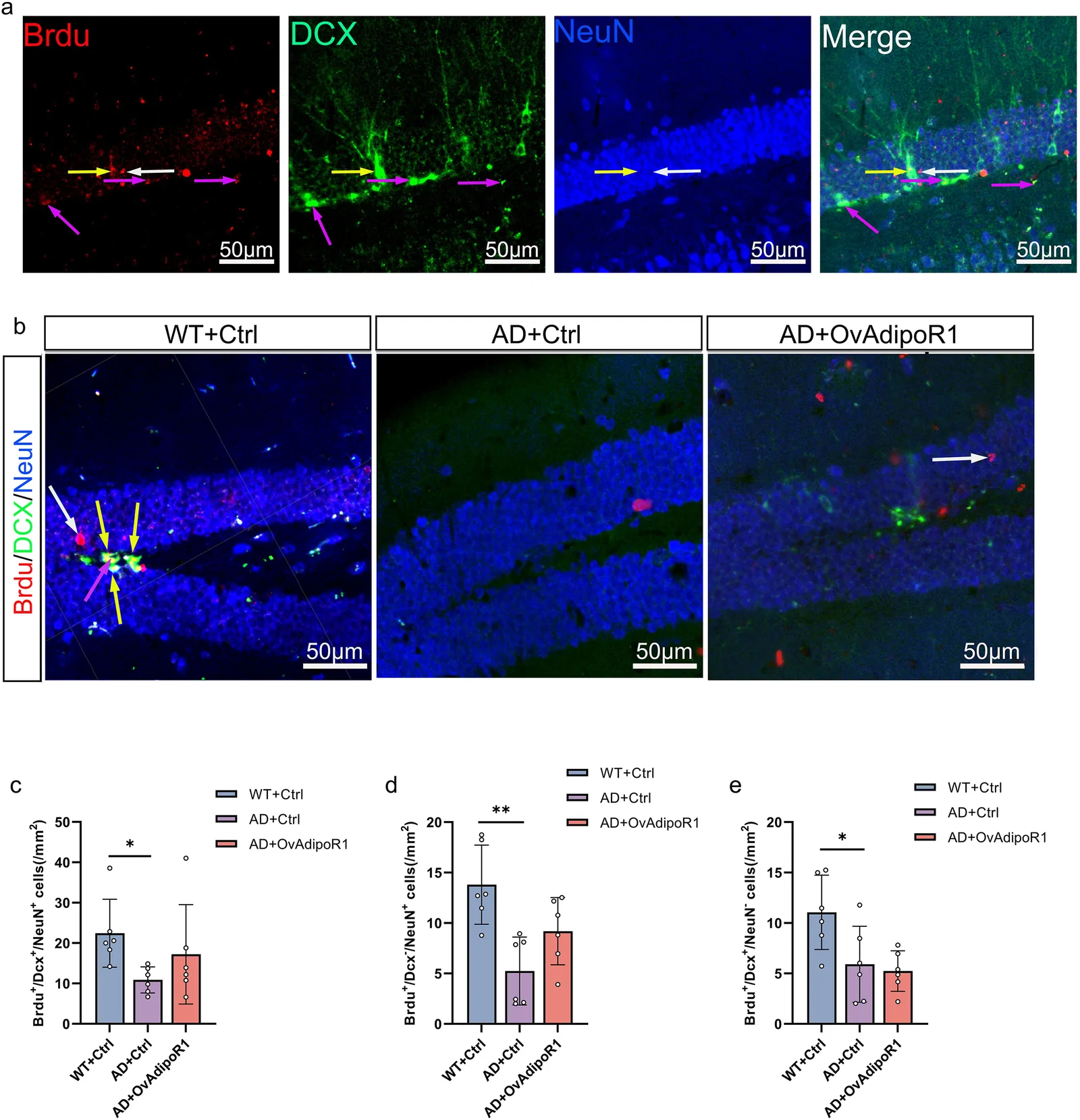
Effect of AdipoR1 on AHN in AD mice. (a) Representative merged image showing cell types. (a) Purple arrow: BrdU^+^/DCX^+^/NeuN^−^ cell (red BrdU^+^, green DCX^+^); White arrow: BrdU^+^/DCX^−^/NeuN^+^ cell (red BrdU^+^, blue NeuN^+^); Yellow arrow: BrdU^+^/DCX^+^/NeuN^+^ cell (red BrdU^+^, green DCX^+^, blue NeuN^+^), scale bar = 50 μm. (b) Representative images of newborn neurons in the hippocampal DG of the three groups, scale bar = 50 μm. (c–e) Density of BrdU^+^/DCX^+^/NeuN^+^ cells, BrdU^+^/DCX^−^/NeuN^+^ cells, and BrdU^+^/DCX^+^/NeuN^−^ cells in the hippocampal DG of each group (n = 6 per group). Data are shown as mean ± SD. ∗*P* < 0.05; ∗∗*P* < 0.01; ∗∗∗*P* < 0.001.

### Overexpression of hippocampal AdipoR1 inhibited NLRP3-mediated neuronal pyroptosis in APP/PS1 mice

Having found that AdipoR1 overexpression ameliorated hippocampal neuronal loss, we investigated whether this recovery was related to pyroptosis. We examined changes in neuronal pyroptosis in the hippocampus of AD + OvAdipoR1 mice. Immunofluorescence co-staining for gasdermin D (GSDMD) and NeuN revealed a significant increase in GSDMD^+^/NeuN^+^ cells in the DG, CA1, and CA3 regions of AD + Ctrl mice compared to WT + Ctrl mice (*P* < 0.001, *P* < 0.001, *P* < 0.001; Fig. 8c). AdipoR1 overexpression significantly reduced the number of GSDMD^+^/NeuN^+^ cells in all three subregions of AD+OvAdipoR1 mice compared to AD + Ctrl mice (*P* = 0.007, *P* = 0.006, *P* < 0.001; Fig. 8c). To confirm whether NLRP3-mediated pyroptosis was inhibited by AdipoR1 overexpression, we examined NLRP3 expression in hippocampal neurons using immunofluorescence. The results showed a significant increase in NLRP3^+^/NeuN^+^ cells in the DG and CA1 regions of AD+Ctrl mice compared to WT + Ctrl mice (*P* = 0.009, *P* = 0.025; Fig. 8d). AdipoR1 overexpression significantly decreased the number of NLRP3^+^/NeuN^+^ cells in these regions of AD+OvAdipoR1 mice compared to AD + Ctrl mice (*P* = 0.006, *P* = 0.040; Fig. 8d). Furthermore, Western blot analyses demonstrated that compared to WT + Ctrl mice, AD + Ctrl mice had significantly increased protein expression levels of NLRP3, GSDMD-C, GSDMD-N, apoptosis-associated speck-like protein containing a CARD (ASC), and IL-1β in the hippocampus (*P* = 0.024, *P* = 0.005, *P* = 0.004, *P* = 0.049, *P* = 0.016; Fig. 8e-f). Overexpression of AdipoR1 significantly decreased the protein levels of NLRP3, GSDMD-C, GSDMD-N, ASC, IL-1β, and caspase-1 in the hippocampus of APP/PS1 mice compared to AD + Ctrl mice (*P* = 0.001, *P* < 0.001, *P* < 0.001, *P* = 0.011, *P* = 0.019, *P* = 0.011; Fig. 8e-f). These data indicated that AdipoR1 overexpression inhibited the activation of NLRP3-mediated neuronal pyroptosis.

**Fig. 8:**
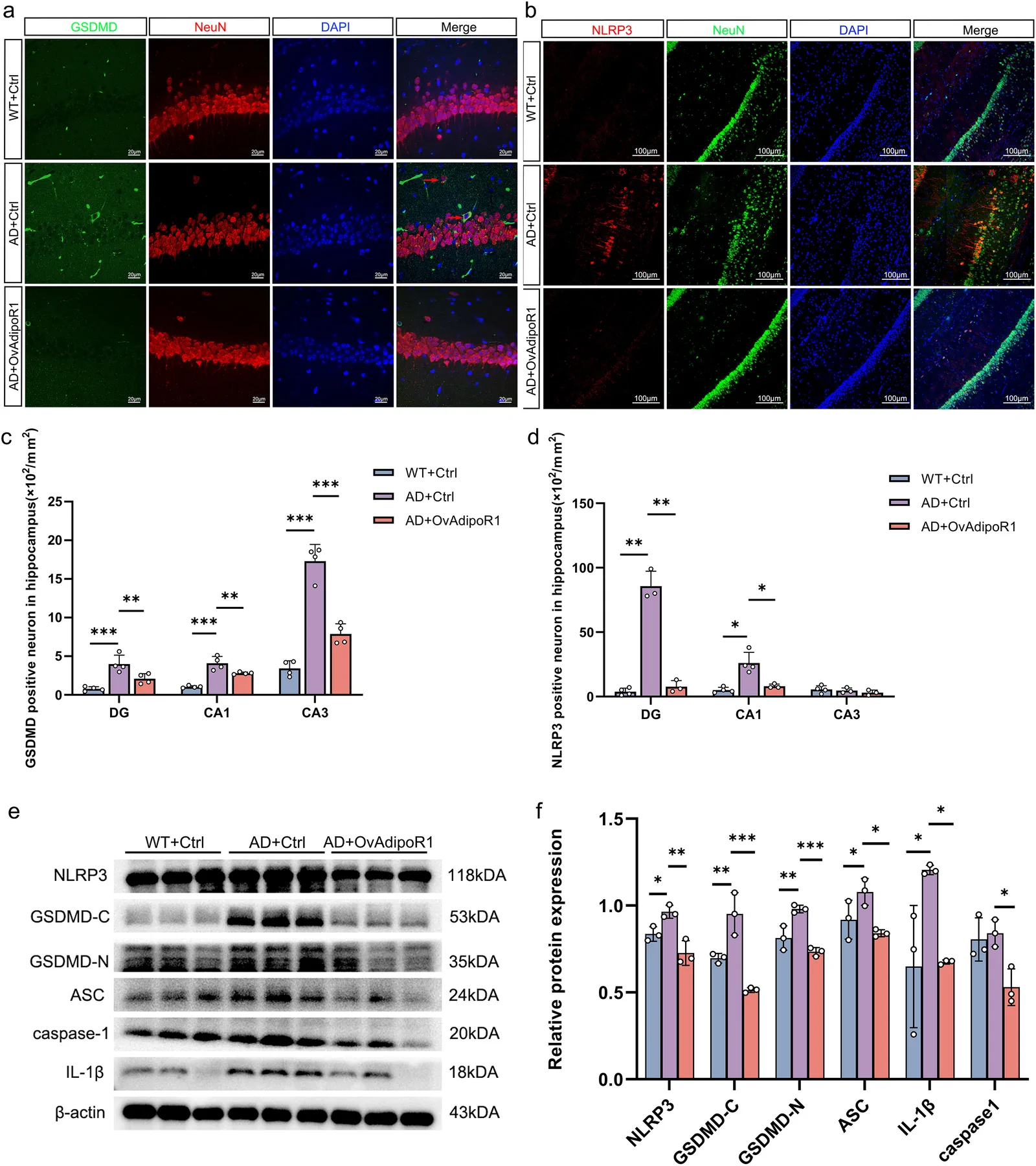
Effect of AdipoR1 on hippocampal neuronal pyroptosis in APP/PS1 mice. (a) Representative immunofluorescence images of GSDMD (green), NeuN (red), and DAPI (blue) in the hippocampus, scale bar = 20 μm. (b) Representative immunofluorescence images of NLRP3 (red), NeuN (green), and DAPI (blue) in the hippocampus, scale bar = 100 μm. (c) Quantification of GSDMD and NeuN co-localization (mean ± SD). (d) Quantification of NLRP3 and NeuN co-localization (mean ± SD). (e–f) Protein expression levels of NLRP3, GSDMD-C, GSDMD-N, ASC, caspase-1, and IL-1β in the hippocampus of the three groups (mean ± SD). ∗*P* < 0.05; ∗∗*P* < 0.01; ∗∗∗*P* < 0.001.

## Discussion

Numerous studies have confirmed that cognitive decline in AD is closely associated with hippocampal neuronal damage and synaptic structural abnormalities [5,40]. However, the molecular mechanisms underlying the progressive and sustained loss of neurons and synapses remain incompletely elucidated. In this study, we found that AdipoR1 expression was significantly downregulated in the hippocampus of APP/PS1 model mice exhibiting cognitive impairment. Notably, regional overexpression of hippocampal AdipoR1 via AAV significantly ameliorated deficits in spatial learning and memory, and effectively attenuated the loss of hippocampal neurons and dendritic spines. More critically, our findings further revealed that the neuroprotective effect mediated by AdipoR1 was not primarily dependent on promoting adult hippocampal neurogenesis, but rather was exerted through the suppression of neuronal pyroptosis. Mechanistic investigations demonstrated that upregulation of AdipoR1 reduced NLRP3 inflammasome activation, thereby attenuating pyroptotic damage in hippocampal tissue and preserving neuronal structure and function. Collectively, these findings uncovered a novel neuroprotective role of AdipoR1 in AD-related neuronal damage, suggesting that the AdipoR1/NLRP3 signaling may be an important molecular node regulating AD neuronal damage. These results might provide novel experimental evidence for understanding the molecular mechanisms underlying hippocampal neuronal loss and for exploring early intervention strategies in AD.

This study revealed the critical role of hippocampal AdipoR1 signaling imbalance in AD-related cognitive impairment and established a direct causal link between regional AdipoR1 upregulation and cognitive functional improvement. Although previous studies have demonstrated that systemic activation of AdipoR signaling (e.g., administration of exogenous AdipoRon) ameliorates cognitive deficits and attenuates neuronal and synaptic damage in AD model mice [41], such interventions cannot distinguish the independent contributions of central versus peripheral AdipoR signaling. Moreover, factors including the pharmacokinetic properties of these agents (e.g., half-life) [42] and off-target effects limit precise resolution of specific receptor subtype alterations in the brain and their pathological significance. Clinical studies have shown that AdipoR1 expression levels are significantly reduced in the hippocampus of AD patients [23], suggesting that hippocampal AdipoR1 signaling dysfunction may contribute to AD-associated neuronal functional impairment. However, merely elevating peripheral adiponectin levels may be insufficient to effectively restore AdipoR1 functional deficiency under AD pathological conditions. Therefore, the present study employed an AAV-mediated hippocampal region-specific AdipoR1 overexpression approach to directly examine the impact of enhanced hippocampal AdipoR1 signaling on cognitive function in APP/PS1 mice. Unlike previous investigations that primarily focused on the effects of systemic AdipoR activation on overall AD pathological progression, metabolic homeostasis, and Aβ/tau protein deposition [41,42], this study, through precise regional manipulation, further elucidated the direct role of this receptor subtype in the modulation of AD cognitive function. Through Morris water maze behavioral testing, we found that hippocampal region-specific AdipoR1 overexpression significantly ameliorated spatial learning and memory impairment in APP/PS1 mice, indicating that hippocampal AdipoR1 functional deficiency has significant pathological implications in AD, and that local restoration of this signaling alone can produce cognitive protective effects. By minimizing potential confounding factors such as peripheral metabolic changes, this study further expanded our understanding of the mechanisms by which AdipoR signaling participated in the regulation of AD cognitive impairment, providing novel theoretical evidence for elucidating hippocampal AdipoR1-mediated neuroprotective mechanisms and for subsequent exploration of precision intervention strategies.

The hippocampus, a critical brain region for memory and cognitive function, relies on the integrity of its neurons to maintain cognitive function [5]. Studies have shown that hippocampal neurons contribute to the formation of episodic memory and spatial navigation through their synaptic plasticity, and their normal electrical activity is fundamental to learning processes [43,44]. In AD pathology, Aβ deposition and tau hyperphosphorylation directly lead to hippocampal neuronal dysfunction and loss. This reduction in neuronal number, along with synaptic damage, is highly correlated with the degree of memory decline in patients [45]. Clinical evidence indicated that metabolic activity was decreased in hippocampal neurons early in AD, and this change preceded the significant worsening of clinical symptoms [46]. Notably, the decline in neurogenic capacity within the hippocampal dentate gyrus is also considered an important factor in the reduction of cognitive reserve in AD patients [47]. However, effective treatments to prevent the abnormal loss of hippocampal neurons in AD patients are still lacking. The adiponectin signaling pathway has been demonstrated to have potential neuroprotective effects [20,23]. Consistent with findings from Ali and Pratap, we found that AdipoR1 expression levels were significantly lower in the hippocampus of AD mice compared to normal mice, and AdipoR1 expression within hippocampal neurons was also markedly decreased [23,48]. Although Liu et al. [26] explored the effect of AdipoRon, a broad agonist of adiponectin receptors, on neural stem cell proliferation in the hippocampus of APP/PS1 transgenic mice, they did not provide specific data on the role of reduced hippocampal AdipoR1 expression in neuronal loss during AD progression. To further investigate the impact of AdipoR1 on hippocampal neurons in AD, we systematically studied the number of neurons and dendritic spines in the DG, CA1, and CA3 regions, as well as hippocampal volume and Aβ deposition, following targeted overexpression of hippocampal AdipoR1. Using stereological methods to accurately quantify neurons and dendritic spines in hippocampal subregions of APP/PS1 mice, our results demonstrated that overexpression of hippocampal AdipoR1 significantly ameliorated hippocampal volume atrophy, neuron loss and dendritic spine loss in the hippocampus of AD mice.

What might be the mechanism by which AdipoR1 rescues the loss of hippocampal neurons in AD mice? We investigated this issue from two perspectives: decreased neurogenesis and increased cell death. The DG is one of the few brain regions where neurogenesis persists into adulthood. Studies have found significantly reduced AHN even in the early or prodromal stages of AD patients and animal models [7,8]. Liu et al. [26] reported that treating APP/PS1 mice with AdipoRon rescued impaired neural stem cell proliferation in the DG. Therefore, we hypothesized that enhanced neurogenesis in the DG might contribute to the reduced loss of neurons across hippocampal subregions in AD mice. Our previous study clearly classified newborn neurons in the hippocampal DG into three types: immature newborn neurons (BrdU^+^/DCX^+^/NeuN^−^), mature newborn neurons (BrdU^+^/DCX^−^/NeuN^+^), and a transitional population (BrdU^+^/DCX^+^/NeuN^+^) [49]. We quantified these populations and found that hippocampal AdipoR1 overexpression did not significantly ameliorate the reduction in any of these three types of newborn neurons in AD mice. Possible reasons for the discrepancy with the findings of Liu et al. [26] include the following. First, the intervention methods differed: they used AdipoRon, a broad agonist of adiponectin receptors, which crosses the blood-brain barrier after oral or intraperitoneal administration and acts widely in the central nervous system, rather than specifically overexpressing AdipoR1 in the hippocampus. Second, the effects of AdipoRon, as a broad agonist, in the hippocampus may be dose-dependent. An earlier study by Lee et al. [50] evaluated the effects of different doses of AdipoRon on adult hippocampal neurogenesis in mice, finding that low-dose AdipoRon significantly promoted hippocampal cell proliferation, whereas high-dose AdipoRon produced the opposite effect, inhibiting cell proliferation and neuronal differentiation. Finally, the BrdU labeling protocol differed; Liu et al. used a 24-h intervention with injections every 6 h, whereas we used daily injections for one week followed by a one-month survival period. Liu et al. labeled proliferating cells within a 24-h window, whereas we tracked long-term survival and differentiation.

For decades, neuroinflammation has been widely recognized as a key driver in the pathogenesis of AD [51,52]. In the AD brain, Aβ deposition triggers excessive microglial activation, resulting in the release of pro-inflammatory cytokines—including IL-1β, TNF-α, and IL-6—that subsequently exacerbate neuronal damage and cognitive decline [52,53]. The adiponectin/AdipoR1 signaling axis has emerged as a critical regulator of neuroinflammatory responses [54,55]. Pharmacological activation of adiponectin receptors by AdipoRon exerts potent anti-inflammatory effects through diverse signaling pathways, notably involving AMPK and PPAR-α activation [42,56]. Moreover, endogenous APN/AdipoR1 signaling enhances mitochondrial function and promotes autophagy-dependent clearance of damaged organelles and misfolded proteins, thereby alleviating the inflammatory burden under pathological conditions [20]. Therefore, targeting AdipoR1 may represent a potential therapeutic strategy to mitigate AD progression by inhibiting hippocampal neuroinflammation. However, how AdipoR1 affects neuroinflammation-mediated death of hippocampal neurons in AD has not been reported. Inflammasomes, as crucial components of the innate immune system, are protein complexes that sense pathogen-associated molecular patterns and damage-associated molecular patterns, leading to the activation of caspase-1, the maturation and release of pro-inflammatory cytokines such as interleukin-1β and interleukin-18, and the induction of inflammatory responses and pyroptosis [57]. Among the various inflammasomes, the NOD-like receptor family pyrin domain-containing protein 1 (NLRP1) and protein 3 (NLRP3) are widely expressed in the central nervous system and have been implicated in AD pathology [57,58]. Traditionally, NLRP1 was thought to be primarily expressed in neurons, while NLRP3 was considered mainly expressed in immune cells such as microglia [59,60]. However, our immunofluorescence staining for NeuN and NLRP3 detected a substantial number of NLRP3-positive neurons in the DG and CA1 regions of APP/PS1 mouse hippocampus. Consistent with our findings, Yue et al. [61] reported that in the temporal cortex of epilepsy patients, NLRP3 inflammasome components were not only localized in astrocytes and microglia but also clearly present in neurons, as shown by immunofluorescence. Additionally, Yao et al. [62] found NLRP3 expression in neurons in both AD animal models and cellular models, and demonstrated that neuronal NLRP3 could regulate tau protein phosphorylation. These studies suggested that neurons may not merely be passive victims of inflammation; under specific conditions, they can actively participate in local inflammatory responses by activating the NLRP3 inflammasome, thereby influencing their own fate and that of surrounding cells. The study by Yang et al. [63] supported this notion, showing that knocking down PHF2 in neurons of 5xFAD mice not only reduced the expression of inflammatory genes but also significantly decreased the activation of microglia and astrocytes. The NLRP3 inflammasome is a core component of the canonical pyroptosis signaling pathway [28]. Previous studies have confirmed that the NLRP3 inflammasome and its mediated pyroptosis are activated in the hippocampus of AD patients and animal models and are closely associated with cognitive impairment [30]. Moreover, Wang et al. [27] pointed out that adiponectin and its homologs could influence the activation of pyroptosis-related factors such as NLRP3 and IL-1β in chondrocytes and epithelial cells via the AdipoR1 pathway, reducing inflammatory responses. Therefore, we hypothesized that AdipoR1 might play an important role in NLRP3-mediated neuronal pyroptosis in AD. In the present study, using immunofluorescence and Western blotting, we analyzed the expression levels of NLRP3-mediated pyroptosis-related proteins, including GSDMD-N, GSDMD-C, ASC, IL-1β, and caspase-1, and found that AdipoR1 effectively inhibited the pyroptosis process during AD pathogenesis. To our knowledge, this is the first study to demonstrate that hippocampal AdipoR1 overexpression can significantly inhibit NLRP3 activation in hippocampal neurons, thereby reducing hippocampal neuronal pyroptosis and ameliorating cognitive decline in an AD animal model.

Although the present study demonstrated that hippocampal overexpression of AdipoR1 could ameliorate cognitive dysfunction and attenuate neuronal damage in APP/PS1 mice, its further clinical translation necessitates exploration in conjunction with more feasible intervention strategies. In this study, AAV-mediated hippocampal AdipoR1 overexpression was employed as an experimental manipulation to investigate the role of AdipoR1 in regulating NLRP3-mediated neuronal pyroptosis and influencing neuronal survival during AD pathogenesis. Given that current intracerebral gene delivery still relies on invasive stereotaxic injection, and that factors such as gene regulation efficiency, tissue distribution, and potential off-target effects may limit its clinical application, direct translation of this strategy to AD patients remains challenging [35,64]. Moving forward, more clinically tractable approaches may involve engineering blood–brain barrier–penetrant AdipoR1-selective agonists, fine-tuning downstream signaling cascades, or constructing neuron-specific delivery vehicles to recapitulate the protective phenotypes observed here within safer, more precisely controlled therapeutic frameworks. However, the principal contribution of this study lies in revealing that AdipoR1/NLRP3 axis may serve as a critical molecular node in modulating NLRP3 activation and neuronal pyroptosis, thereby underscoring the potential therapeutic value of AdipoR1 signaling modulation. Future studies should further elucidate feasible approaches to modulate AdipoR1 signaling, including the exploration of pharmacological strategies to enhance endogenous AdipoR1 function and the dissection of its downstream protective signaling networks, thereby providing a theoretical foundation for the development of more clinically viable intervention strategies for AD.

A limitation of this study is the use of only the Morris water maze to assess cognitive function, resulting in relatively simple behavioral data. Although the water maze is considered the “gold standard" in AD research, it primarily targets hippocampus-dependent spatial memory. Moreover, we cannot exclude the possibility that anxiety-like behaviors may act as a confounding factor, potentially interfering with the assessment of cognitive function. Alzheimer’s disease is a complex neurodegenerative disorder that impairs multiple cognitive domains. In the future, we need to incorporate a broader range of behavioral tests to obtain more robust and comprehensive results. Furthermore, the interventions and experiments in this study were conducted solely in an animal model; we lack in vitro cellular validation of the causal relationship among AdipoR1 intervention in hippocampal neurons, AD-related pathological changes, and NLRP3-mediated pyroptosis.

## Author contributions

CH: Participated in the research, analysis, and writing of the experiments. JL, LJ, YZ, LZ, FC, WY: Guided the experiments and provided technical assistance. JL, YZ, SW, QP: Assisted in animal model construction and sample preparation. YL, JT, YD, YQ, QX: Helped with the project design. YT, CZ: Writing – review and editing, supervision, resources, project administration, funding acquisition, and conceptualization.

## Declaration of Interest Statement

The authors report no biomedical financial interests or potential conflicts of interest.

## Figures and Tables

**figs1:**
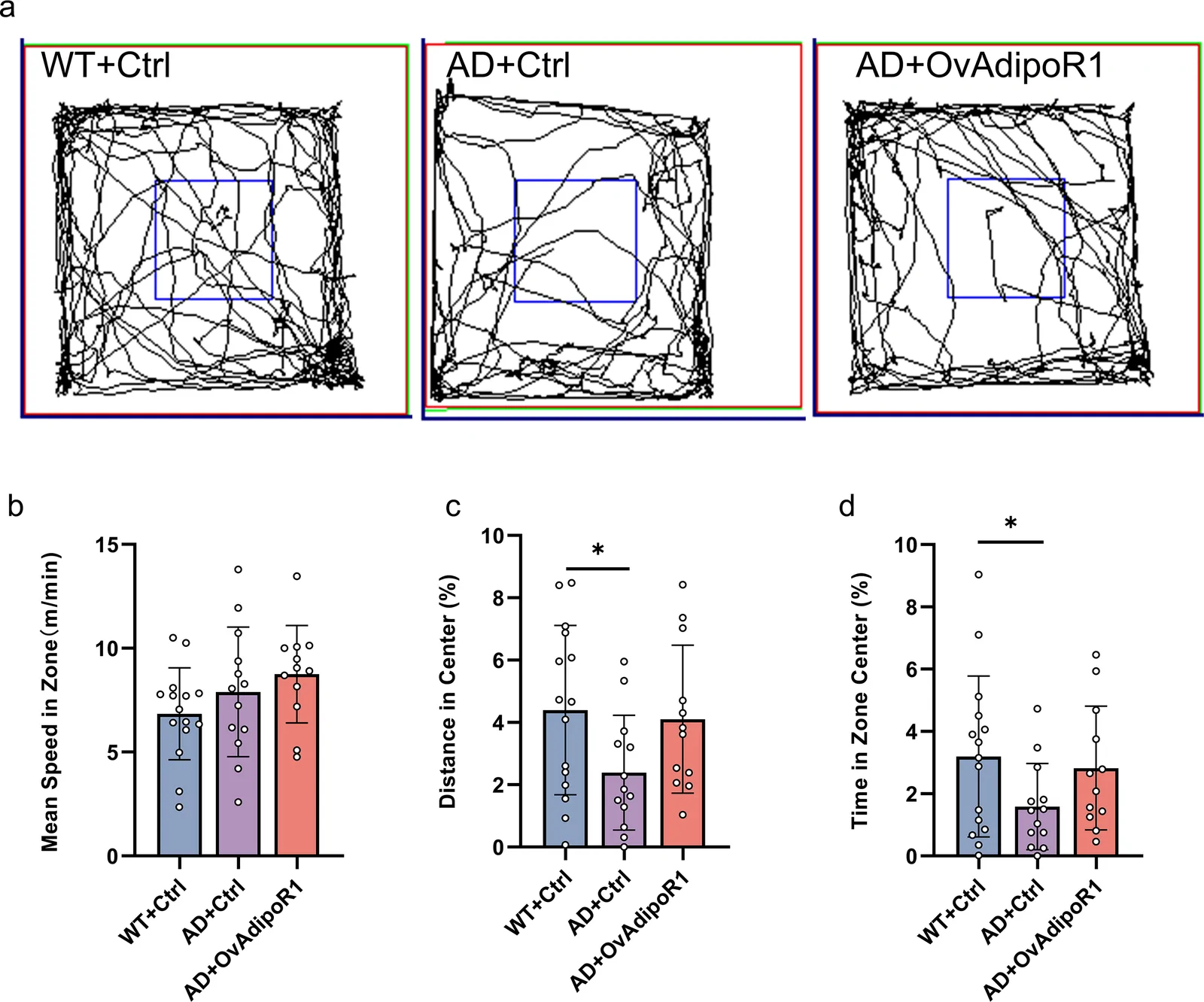


## References

[1] Scheltens P., De Strooper B., Kivipelto M., Holstege H., Chételat G., Teunissen C.E. (2021). Alzheimer’s disease. Lancet.

[2] Zheng Q., Wang X. (2025). Alzheimer’s disease: insights into pathology, molecular mechanisms, and therapy. Protein Cell.

[3] Hyman B.T., Van Hoesen G.W., Damasio A.R., Barnes C.L. (1984). Alzheimer’s disease: cell-specific pathology isolates the hippocampal formation. Science.

[4] Maleki Balajoo S., Eickhoff S.B., Masouleh S.K., Plachti A., Waite L., Saberi A. (2023). Hippocampal metabolic subregions and networks: behavioral, molecular, and pathological aging profiles. Alzheimer’s Dement.

[5] West M.J., Coleman P.D., Flood D.G., Troncoso J.C. (1994). Differences in the pattern of hippocampal neuronal loss in normal ageing and alzheimer’s disease. Lancet.

[6] Gómez-Isla T., Hollister R., West H., Mui S., Growdon J.H., Petersen R.C. (1997). Neuronal loss correlates with but exceeds neurofibrillary tangles in alzheimer’s disease. Ann Neurol.

[7] Salta E., Lazarov O., Fitzsimons C.P., Tanzi R., Lucassen P.J., Choi S.H. (2023). Adult hippocampal neurogenesis in alzheimer’s disease: a roadmap to clinical relevance. Cell Stem Cell.

[8] Geigenmüller J.N., Tari A.R., Wisloff U., Walker T.L. (2024). The relationship between adult hippocampal neurogenesis and cognitive impairment in alzheimer’s disease. Alzheimer’s Dement.

[9] Kumari S., Dhapola R., Sharma P., Paidlewar M., Vellingiri B., Medhi B. (2025). Unravelling neuronal death mechanisms: the role of cytokines and chemokines in immune imbalance in alzheimer’s disease progression. Ageing Res Rev.

[10] Kempermann G., Jessberger S., Steiner B., Kronenberg G. (2004). Milestones of neuronal development in the adult hippocampus. Trends Neurosci.

[11] Choi S.H., Bylykbashi E., Chatila Z.K., Lee S.W., Pulli B., Clemenson G.D. (2018). Combined adult neurogenesis and BDNF mimic exercise effects on cognition in an Alzheimer’s mouse model. Science.

[12] Li Y.D., Luo Y.J., Xie L., Tart D.S., Sheehy R.N., Zhang L. (2023). Activation of hypothalamic-enhanced adult-born neurons restores cognitive and affective function in alzheimer’s disease. Cell Stem Cell.

[13] Walgrave H., Balusu S., Snoeck S., Vanden Eynden E., Craessaerts K., Thrupp N. (2021). Restoring miR-132 expression rescues adult hippocampal neurogenesis and memory deficits in alzheimer’s disease. Cell Stem Cell.

[14] Song R., Yin S., Wu J., Yan J. (2025). Neuronal regulated cell death in aging-related neurodegenerative diseases: key pathways and therapeutic potentials. Neural Regen Res.

[15] Rui W., Xiao H., Fan Y., Ma Z., Xiao M., Li S. (2021). Systemic inflammasome activation and pyroptosis associate with the progression of amnestic mild cognitive impairment and alzheimer’s disease. J Neuroinflammation.

[16] Moonen S., Koper M.J., Van Schoor E., Schaeverbeke J.M., Vandenberghe R., Von Arnim C.A.F. (2023). Pyroptosis in alzheimer’s disease: cell type-specific activation in microglia, astrocytes and neurons. Acta Neuropathol.

[17] Cookson B.T., Brennan M.A. (2001). Pro-inflammatory programmed cell death. Trends Microbiol.

[18] Liu Y., Pan R., Ouyang Y., Gu W., Xiao T., Yang H. (2024). Pyroptosis in health and disease: mechanisms, regulation and clinical perspective. Signal Transduct Targeted Ther.

[19] Zhu P., Luo Y., Li Y., Tang J., Liu L., Deng Y. (2025). Downregulation of AdipoR1 in the hippocampus impairs synaptic function and structure and causes depression-like behavior. Transl Psychiatry.

[20] Zhang S., Wu X., Wang J., Shi Y., Hu Q., Cui W. (2022). Adiponectin/AdipoR1 signaling prevents mitochondrial dysfunction and oxidative injury after traumatic brain injury in an SIRT3-dependent manner. Redox Biol.

[21] Thundyil J., Pavlovski D., Sobey C.G., Arumugam T.V. (2012). Adiponectin receptor signalling in the brain. Br J Pharmacol.

[22] Thundyil J., Tang S.C., Okun E., Shah K., Karamyan V.T., Li Y.I. (2010). Evidence that adiponectin receptor 1 activation exacerbates ischemic neuronal death. Exp Transl Stroke Med.

[23] Ali T., Rehman S.U., Khan A., Badshah H., Abid N.B., Kim M.W. (2021). Adiponectin-mimetic novel nonapeptide rescues aberrant neuronal metabolic-associated memory deficits in alzheimer’s disease. Mol Neurodegener.

[24] Jian Y., Yuan S., Yang J., Lei Y., Li X., Liu W. (2022). Aerobic exercise alleviates abnormal autophagy in brain cells of APP/PS1 mice by upregulating AdipoR1 levels. Int J Mol Sci.

[25] Feng J., Ma X., Liu Y., Shi X., Jin L., Le Y. (2024). The role of human adiponectin receptor 1 in 2-ethylhexyl diphenyl phosphate induced lipid metabolic disruption. Environ Sci Technol.

[26] Liu B., Liu J., Wang J., Liu C., Yan H. (2020). AdipoRon improves cognitive dysfunction of alzheimer’s disease and rescues impaired neural stem cell proliferation through AdipoR1/AMPK pathway. Exp Neurol.

[27] Wang B., Li Y., Hu S., Peng K. (2022). C1q/tumor necrosis factor-related protein 9 protects cultured chondrocytes from IL-1β induced inflammatory injury by inhibiting NLRP3 inflammasome activation via the AdipoR1/AMPK axis. Environ Toxicol.

[28] Han Y.H., Liu X.D., Jin M.H., Sun H.N., Kwon T. (2023). Role of NLRP3 inflammasome-mediated neuronal pyroptosis and neuroinflammation in neurodegenerative diseases. Inflamm Res.

[29] Heneka M.T., McManus R.M., Latz E. (2018). Inflammasome signalling in brain function and neurodegenerative disease. Nat Rev Neurosci.

[30] McManus R.M., Latz E. (2024). NLRP3 inflammasome signalling in alzheimer’s disease. Neuropharmacology.

[31] Radde R., Bolmont T., Kaeser S.A., Coomaraswamy J., Lindau D., Stoltze L. (2006). Abeta42-driven cerebral amyloidosis in transgenic mice reveals early and robust pathology. EMBO Rep.

[32] Wang C., Gao W.R., Yin J., Wang Z.J., Qi J.S., Cai H.Y. (2021). Chronic sleep deprivation exacerbates cognitive and synaptic plasticity impairments in APP/PS1 transgenic mice. Behav Brain Res.

[33] Chao F.L., Zhang Y., Zhang L., Jiang L., Zhou C.N., Tang J. (2021). Fluoxetine promotes hippocampal oligodendrocyte maturation and delays learning and memory decline in APP/PS1 mice. Front Aging Neurosci.

[34] Jiang L., Ma J., Zhang Y., Zhou C.N., Zhang L., Chao F.L. (2018). Effect of running exercise on the number of the neurons in the hippocampus of young transgenic APP/PS1 mice. Brain Res.

[35] Ling Q., Herstine J.A., Bradbury A., Gray S.J. (2023). AAV-Based in vivo gene therapy for neurological disorders. Nat Rev Drug Discov.

[36] Wang Y., Zhou Y., Wang S., Liu L., Jiang L., Zhang Y. (2025). Voluntary wheel exercise improves learning and memory impairment caused by hippocampal Hb-α deficiency by reducing microglial activation and reversing synaptic damage. Brain Behav Immun.

[37] Challis R.C., Ravindra Kumar S., Chan K.Y., Challis C., Beadle K., Jang M.J. (2019). Systemic AAV vectors for widespread and targeted gene delivery in rodents. Nat Protoc.

[38] Tang Y., Janssen W.G., Hao J., Roberts J.A., McKay H., Lasley B. (2004). Estrogen replacement increases spinophilin-immunoreactive spine number in the prefrontal cortex of female rhesus monkeys. Cerebr Cortex.

[39] Hao J., Janssen W.G., Tang Y., Roberts J.A., McKay H., Lasley B. (2003). Estrogen increases the number of spinophilin-immunoreactive spines in the hippocampus of young and aged female rhesus monkeys. J Comp Neurol.

[40] Selkoe D.J. (2002). Alzheimer’s disease is a synaptic failure. Science.

[41] Ng R.C.L., Cheng O.Y., Jian M., Kwan J.S.C., Ho P.W.L., Cheng K.K.Y. (2016). Chronic adiponectin deficiency leads to Alzheimer’s disease-like cognitive impairments and pathologies through AMPK inactivation and cerebral insulin resistance in aged mice. Mol Neurodegener.

[42] Ng R.C.L., Jian M., Ma O.K.F., Bunting M., Kwan J.S.C., Zhou G.J. (2021). Chronic oral administration of adipoRon reverses cognitive impairments and ameliorates neuropathology in an alzheimer’s disease mouse model. Mol Psychiatr.

[43] Buzsáki G., Moser E.I. (2013). Memory, navigation and theta rhythm in the hippocampal-entorhinal system. Nat Neurosci.

[44] Bin Ibrahim M.Z., Goh L.Z.N., Koh N.W.K., Polepalli J.S., Behnisch T., Sajikumar S. (2025). Hippocampal CA2 to CA1: a metaplastic switch for memory encoding. Proc Natl Acad Sci U S A.

[45] Capilla-López M.D., Deprada A., Andrade-Talavera Y., Martínez-Gallego I., Coatl-Cuaya H., Sotillo P. (2025). Synaptic vulnerability to amyloid-β and tau pathologies differentially disrupts emotional and memory neural circuits. Mol Psychiatr.

[46] Bano D., Ehninger D., Bagetta G. (2023). Decoding metabolic signatures in alzheimer’s disease: a mitochondrial perspective. Cell Death Discov.

[47] Moreno-Jiménez E.P., Flor-García M., Terreros-Roncal J., Rábano A., Cafini F., Pallas-Bazarra N. (2019). Adult hippocampal neurogenesis is abundant in neurologically healthy subjects and drops sharply in patients with alzheimer’s disease. Nat Med.

[48] Pratap A.A., Holsinger R.M.D. (2020). Altered brain adiponectin receptor expression in the 5xFAD mouse model of alzheimer’s disease. Pharmaceuticals.

[49] He Q., Jiang L., Zhang Y., Yang H., Zhou C.N., Xie Y.H. (2021). Anti-LINGO-1 antibody ameliorates cognitive impairment, promotes adult hippocampal neurogenesis, and increases the abundance of CB1R-rich CCK-GABAergic interneurons in AD mice. Neurobiol Dis.

[50] Lee T.H., Christie B.R., Van Praag H., Lin K., Siu P.M.F., Xu A. (2021). AdipoRon treatment induces a dose-dependent response in adult hippocampal neurogenesis. Int J Mol Sci.

[51] Akiyama H., Barger S., Barnum S., Bradt B., Bauer J., Cole G.M. (2000). Inflammation and alzheimer’s disease. Neurobiol Aging.

[52] Heneka M.T., Carson M.J., El Khoury J., Landreth G.E., Brosseron F., Feinstein D.L. (2015). Neuroinflammation in alzheimer’s disease. Lancet Neurol.

[53] Leng F., Edison P. (2021). Neuroinflammation and microglial activation in alzheimer disease: where do we go from here?. Nat Rev Neurol.

[54] Nicolas S., Cazareth J., Zarif H., Guyon A., Heurteaux C., Chabry J. (2017). Globular adiponectin limits microglia pro-inflammatory phenotype through an AdipoR1/NF-κB signaling pathway. Front Cell Neurosci.

[55] Jian M., Kwan J.S., Bunting M., Ng R.C., Chan K.H. (2019). Adiponectin suppresses amyloid-β oligomer (AβO)-induced inflammatory response of microglia via AdipoR1-AMPK-NF-κB signaling pathway. J Neuroinflammation.

[56] Khandelwal M., Manglani K., Upadhyay P., Azad M., Gupta S. (2022). AdipoRon induces AMPK activation and ameliorates alzheimer’s-like pathologies and associated cognitive impairment in APP/PS1 mice. Neurobiol Dis.

[57] Hu B., Zhang J., Huang J., Luo B., Zeng X., Jia J. (2024). NLRP3/1-mediated pyroptosis: beneficial clues for the development of novel therapies for alzheimer’s disease. Neural Regen Res.

[58] Zhang M., Yang L., Li Z., Zhou L., Wang Y., Wang H. (2024). NLRP1 inflammasome in neurodegenerative disorders: from pathology to therapies. Cytokine Growth Factor Rev.

[59] Yao J., Wang Z., Song W., Zhang Y. (2023). Targeting NLRP3 inflammasome for neurodegenerative disorders. Mol Psychiatr.

[60] Vontell R.T., De Rivero Vaccari J.P., Sun X., Gultekin S.H., Bramlett H.M., Dietrich W.D. (2023). Identification of inflammasome signaling proteins in neurons and microglia in early and intermediate stages of alzheimer’s disease. Brain Pathol.

[61] Yue J., Wei Y.J., Yang X.L., Liu S.Y., Yang H., Zhang C.Q. (2020). NLRP3 inflammasome and endoplasmic reticulum stress in the epileptogenic zone in temporal lobe epilepsy: molecular insights into their interdependence. Neuropathol Appl Neurobiol.

[62] Yao J., Li Y., Liu X., Liang W., Li Y., Wu L. (2025). FUBP3 mediates the amyloid-β-induced neuronal NLRP3 expression. Neural Regen Res.

[63] Yang G., Ren Y., Zhong P., Patel P.J., Chen X.Q., Wan L. (2026). Histone demethylase PHF2 regulates inflammatory genes in alzheimer’s disease. Mol Psychiatr.

[64] Rafii M.S., Tuszynski M.H., Thomas R.G., Barba D., Brewer J.B., Rissman R.A. (2018). Adeno-associated viral vector (serotype 2)-nerve growth factor for patients with alzheimer disease: a randomized clinical trial. JAMA Neurol.

