# Supplementary material for: Overexpression of hippocampal AdipoR1 ameliorates cognitive dysfunction in APP/PS1 mice through inhibiting NLRP3-Mediated neuronal pyroptosis
Source: Neurotherapeutics. 2026 Oct 26;24(1):e01093. doi: 10.1016/j.neurot.2026.e01093 (PMC13722108; doi:10.1016/j.neurot.2026.e01093)
Supplement: Supplementary file 1 [file mmc1.docx]

**Title: Overexpression of Hippocampal AdipoR1 Ameliorates Cognitive Dysfunction in APP/PS1 Mice through Inhibiting NLRP3-Mediated Neuronal Pyroptosis**

**Supplemental Information**

**Materials and methods**

Open field test (OFT).

The OFT was conducted to assess spontaneous locomotor activity and anxiety-like behavior in mice. Testing was performed in a quiet behavioral testing room. The open field arena consisted of a custom-made black opaque acrylic chamber (40 × 40 × 40 cm), with the floor virtually divided into a central zone (20 × 20 cm, 25% of the total area) and a peripheral zone. A digital camera was fixed 100 cm above the arena. Prior to testing, mice were transferred to the testing room 1 h in advance for environmental habituation. At the start of the session, each mouse was gently placed in the peripheral zone of the arena facing the wall and allowed to freely explore for 5 min. Motor trajectories were automatically recorded and analyzed using the SMART 3.0 video tracking system (Harvard Apparatus), and the following parameters were quantified: average locomotor speed, percentage of total distance traveled in the center zone, and percentage of total time spent in the center zone. After each trial, the arena floor and walls were thoroughly wiped with 75% ethanol and allowed to dry completely to eliminate residual odor before the next test. All experiments were conducted during a fixed time window (9:00 a.m. to 3:00 p.m.) to minimize circadian variations.

**Supplementary Figure legends**

**Supplementary Fig. 1** Representative locomotor trajectories and quantitative analyses of the open field test in WT+Ctrl, AD+Ctrl, and AD+OvAdipoR1 mice (a) Representative locomotor trajectories in the open field arena. (b) Average movement speed (mean ± SD). (c) Percentage of distance traveled in the center zone relative to total distance (mean ± SD). (d) Percentage of time spent in the center zone relative to total time (mean ± SD).
